# Death from exsanguination due to power drill injuries in a complex suicide: a case report

**DOI:** 10.1007/s12024-023-00595-5

**Published:** 2023-03-02

**Authors:** Živa Ledinek, Peter Kadiš, Tina Čakš Golec

**Affiliations:** 1 Department of Pathology, University Medical Centre Maribor, Ljubljanska ulica 5, 2000 Maribor, Slovenia; 2 Department of Pathology, Faculty of Medicine, Taborska ulica 8, 2000 Maribor, Slovenia; 3 Department of Forensic Medicine, Faculty of Medicine, Taborska ulica 8, 2000 Maribor, Slovenia

**Keywords:** Complex suicide, Power drill, Autopsy, Exsanguination

## Abstract

We present a case of a complex suicide of a 66-year-old man with a history of several psychiatric disorders. He attempted to commit suicide by inflicting cut wounds on his forearms, wrists, and neck but afterwards changed the method of suicide by using an electric power drill. After several unsuccessful attempts to drill a hole in either his head, thorax, or abdomen, he managed to perforate the common carotid artery on the right side of his neck and subsequently died from exsanguination.

## Introduction

Suicides by power tools are very rare and most often attributed to sawing tools [1]. Only a handful of cases describe the use of a power drill as a method of choice. All known cases of suicide by a power drill list cranial or thoracic injuries (or a combination of both) as the cause of death [1–3]. There are also reports of self-inflicted injuries using a power drill without a lethal outcome, with described cranial, cervical, thoracic or abdominal injuries [4, 5].

When describing a suicidal act, the distinction is made between the so-called simple and complex suicides [6, 7]. The latter are defined as a suicide in which more than one suicide method is applied. Complex suicides are rarer and account for 1.5 to 5.6% in different studies [7–9]. Most of them are unplanned when suicide methods are used one after another if the first method does not cause death, if death occurs too slowly, or if the chosen method causes too much pain [7]. Among the most common suicide methods, including the complex suicide cases, are hanging, use of firearms, cut wounds to the neck or wrists, ingestion of pesticides or other pharmacologically active substances, drowning, use of electroshock, or a shot with a slaughterer’s gun [7, 10, 11].

We present a case of a complex suicide with a power drill. This is the first described case in which the cause of death was neither a cranial injury nor an injury to the thoracic organs but exsanguination due to a ruptured carotid artery after a penetrative injury to the neck.

## Case report

**Fig. 1:**
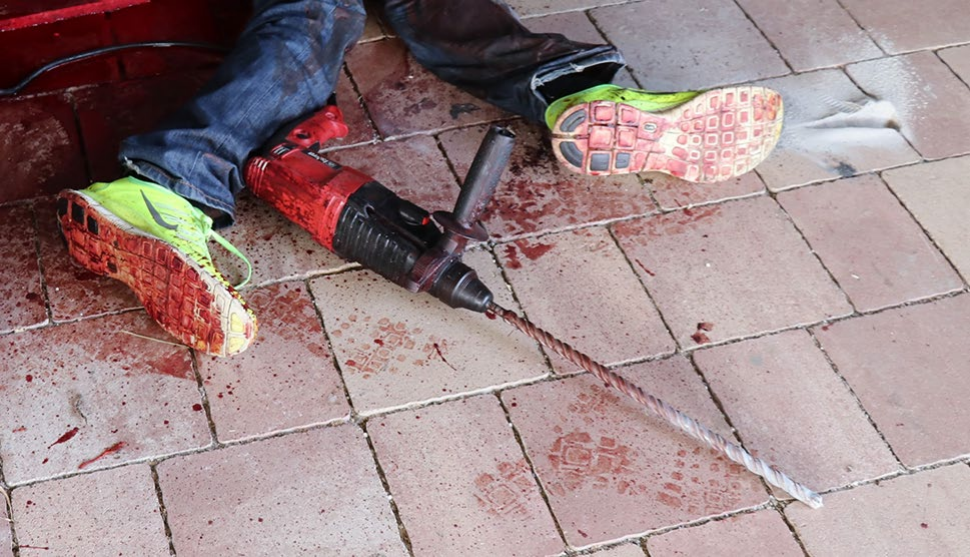
Power drill as found at the scene

A 66-year-old man with known alcohol dependence and epilepsy was found lying on the floor of his garage in the pool of his blood. The coroner concluded that the man committed suicide, using an electric power drill (with an attached 14-mm drill bit at a length of 40 cm) presented in Fig. 1.

**Fig. 2:**
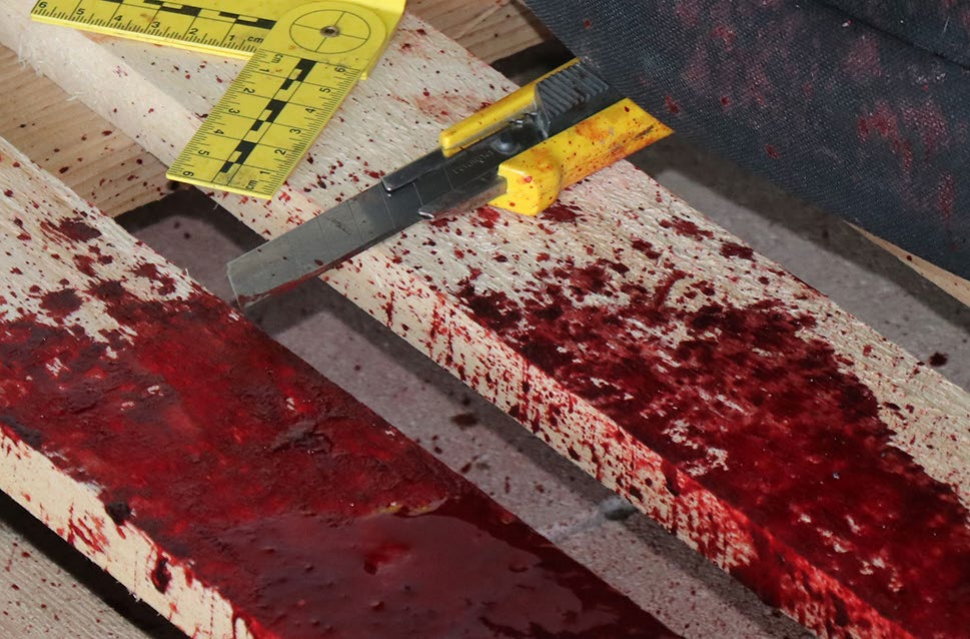
Utility knife as found at the scene

External examination of the body revealed two types of injuries. There were cut wounds from a utility knife with a blade width of 25 mm (Fig. 2) present on both forearms and neck, as well as wounds attributed to the power drill present on the forehead, in the left paraumbilical region of the abdomen, on the right side of the thorax, and the right side of the neck with a penetrative wound extending to the back of the body. The presence of two different types of wounds, caused by two different objects, is indicative of a complex suicide.

Investigation at the death scene showed the deceased lying front face on the floor of the locked garage with a power drill machine by his legs. When the body was found, the power drill machine was still running and has made a hole the in stone paving on the floor (seen in Fig. 1). There was a utility knife found on the left side of the body at the top of a wooden pallet, where it was tossed after an unsuccessful attempt of suicide by cutting the forearms and neck. Bloody handprints were found on the electric cord and power drill machine, indicating that the machine had been chosen as the second method of suicide. Bloody handprints on the machine handle indicated that it had been held by the handle in attempts to drill a hole in either the skull, chest, or abdomen, but the deceased was unable to produce enough force to cause severe internal injuries by holding the machine in this position. Based on the location of the blood pool and the spraying pattern of blood stains on the nearby objects, the deceased most probably placed the drill in an upward position and leaned on the top of it, producing enough force to drill a hole in his cervical vertebrae. To adjust the height of the drill, it was placed on the nearby wooden pallet. When the deceased lost consciousness, he fell forward, the bloody footprints indicated his steps, and the drill was displaced towards his legs.

The cause of death was determined to be external exsanguination due to the injury of the right common carotid artery.

### Clinical history

A revision of the patient’s medical history showed that he suffered from several psychiatric conditions. He had a 27-year-long history of alcohol dependency, which was unsuccessfully managed despite three long-time in-patient treatments, and was diagnosed with a narcissistic personality disorder. There is no record of him having any suicidal thoughts or intents at the time of his follow-up treatments; however, his last psychiatric evaluation was done in 2012; therefore, little is known of his mental state at the time of his death. He was diagnosed with epilepsy after a major head injury he suffered in a car crash in 2009 but was not taking the prescribed medication, although he attended his regular appointments with a neurologist. His medical record was also significant for multiple treatments at the emergency centre after falls due to either an epileptic seizure or alcohol intoxication. The last entry in his medical record was less than a month before his death when he was triaged in the emergency centre for a thumb injury, but he left before receiving medical attention.

### Autopsy report

**Fig. 3:**
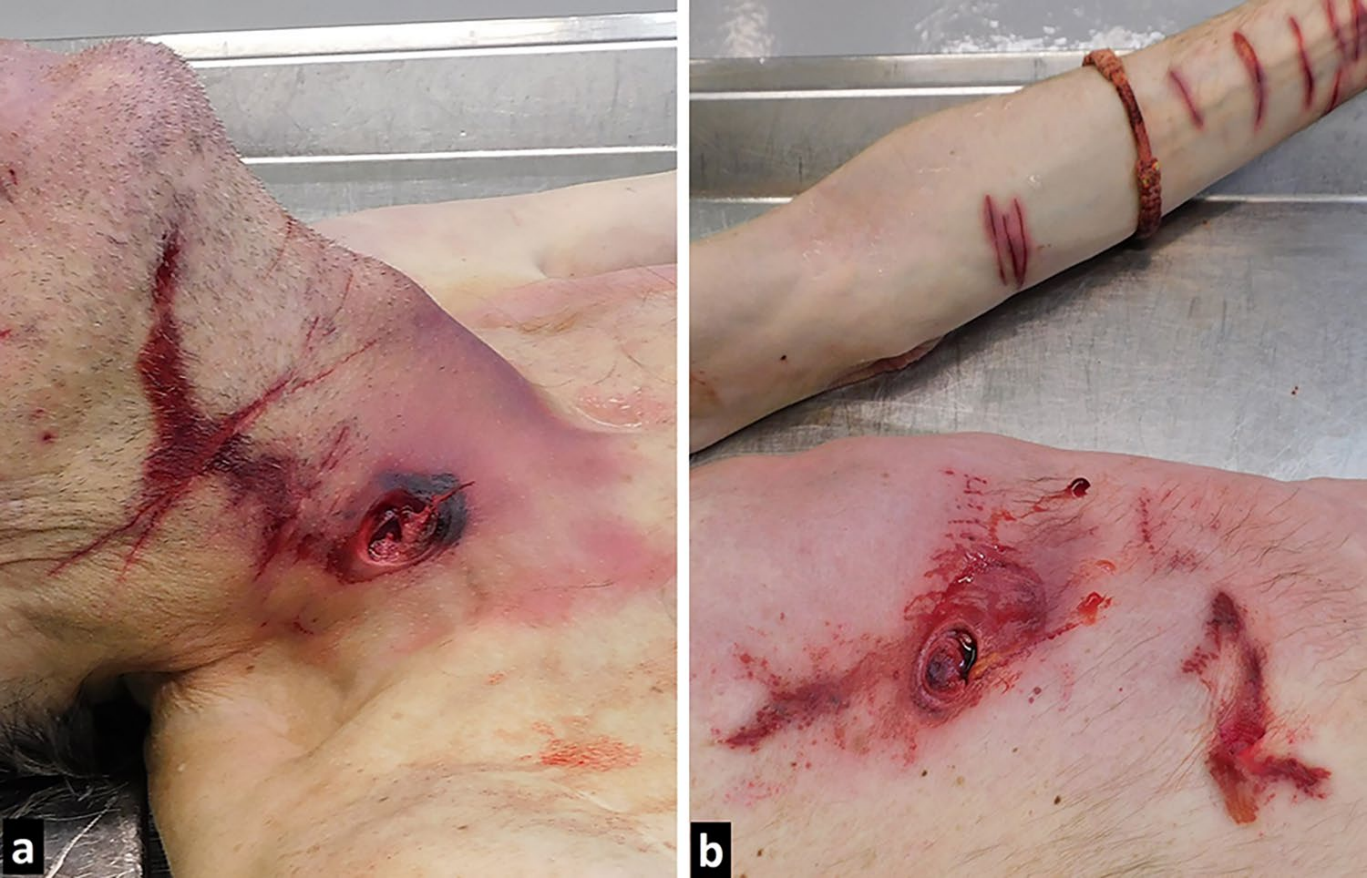
External injuries. **a** Shallow cut wounds from utility knife are seen on the skin of the neck above the penetrative wound. **b** The wound on the abdomen. There is a clear drill pattern visible on the skin above the wound. In the back of the photo, shallow cut wounds are seen on the left forearm

**Fig. 4:**
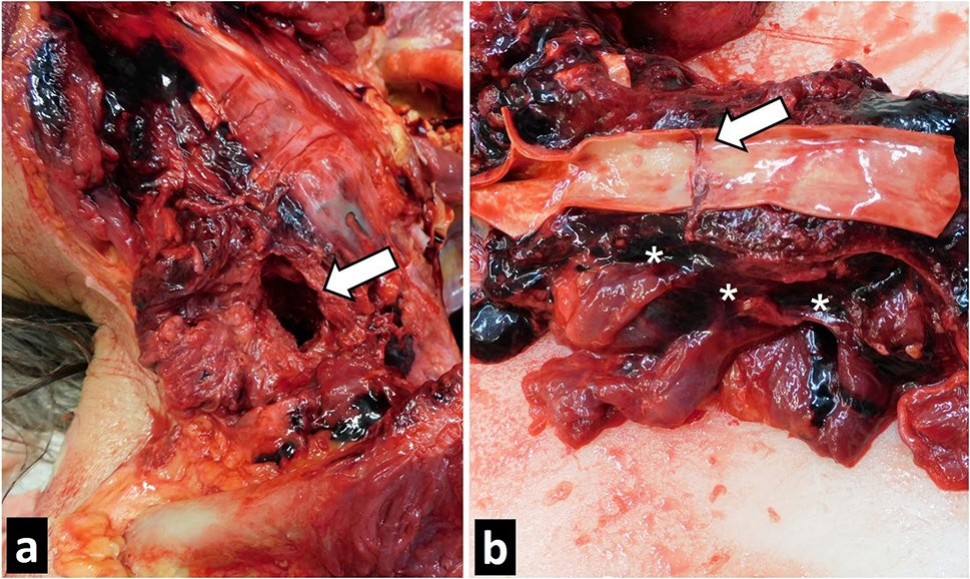
Internal injuries. A penetrative wound on the right side of the neck (**a**) extends through the soft tissues and towards the 4th and 5th cervical vertebrae (arrow). The drill injured the carotid artery (**b**) by causing a laceration in the arterial wall (arrow) and leading to exsanguination with extensive soft tissue hematoma (*)

On external examination, the corpse of a 66-year-old, well-developed, poorly nourished man had circle-shaped skin defect measuring 2 cm in diameter, with regular, slightly serrated edges, present just above the right clavicular bone (Fig. 3a). The defect continued upward as an 8-cm-long abrasion of the skin, measuring from 1.5 to 2 cm in diameter. A bluish bruise was seen on the surface of the skin on the right side of the neck. Beginning on the front side of the neck, the penetrative wound extended through the soft tissues of the neck and the 4th and 5th cervical vertebrae (Fig. 4a). The bone defect measured 1.5 cm. The drill caused two lacerations in the wall of the right common carotid artery (Fig. 4b), each measuring 0.5 cm in diameter. The exit wound was located near the medial rim of the scapula and measured 2 cm in diameter.

Other wounds attributed to the effect of the power drill were present on the head and trunk. There was a circular abrasion of the skin, measuring 2 cm in diameter, present on the forehead near the hairline. There was a 2-cm-long abrasion with a brownish crust on the bottom, running from above the eyebrow towards the circular abrasion. Under the left nipple and towards the anterior axillary line, there was an irregular red-violet abrasion measuring 10 × 15 cm. On the skin of the abdomen, there was a circular defect measuring 2 cm in diameter (Fig. 3b), like the previously described neck wound but without penetration into the abdominal wall. Examination of internal organs revealed minimal bleeding in the peritoneal cavity, located around the spleen and pancreas.

There were eight shallow, transverse cuts on both sides of the neck (Fig. 3a). Cut wounds had sharp edges, measuring from 7 to 10 cm, without signs of active bleeding. Similar wounds, measuring from 2.5 to 6 cm, were present on both forearms (Fig. 3b). They were inflicted with a utility knife (Fig. 2). Scars from previous cuts were visible on the left forearm, suggesting previous episodes of self-harm behaviour, which was not documented in his medical record. Multiple surgical scars were also present on the skin of the trunk, attributed to previously sustained and documented injuries. Lower limbs sustained no injuries and had no changes on the surface of the skin.

### Toxicology report

At the time of autopsy, a blood sample was obtained from the patient and sent for toxicological analysis. Chromatography was performed, but it did not reveal the presence of any pharmacologically active substances, including alcohol (blood level of alcohol was < 0.1 g/l).

## Discussion

Cases of fatal injuries attributed to a power drill are extremely rare. To date, only a handful of case reports describing death from a penetrative wound by a power drill were published [1–3, 12–15]. In most cases, injuries were self-inflicted, and one case was an accidental craniocerebral penetrating wound due to a fall from a stepladder while perforating a ceiling with a drill [12]. So far, injuries from power drills, including the head, were most commonly described, but there are also reports of injuries to the chest or both [2]. Not all the injuries inflicted with a power drill were lethal, and in rare cases, patients recovered completely, even after a brain injury [4]. Byard described a case of a complex suicide with a penetrative wound to the frontal lobe of the brain, where the cause of death was attributed to subsequent stab wounds in the abdomen with a sharp blade [2]. In our case, the deceased attempted to drill a hole in his head, thorax, and abdomen, but neither of those attempts were successful. In all three cases, the wound was superficial, although the wound on the abdomen caused minimal intraabdominal bleeding around the spleen and pancreas without actual injury to the inner organs or soft tissues. The deceased then successfully drilled a hole on the side of his neck, penetrating through the soft tissue and the cervical spine, extending to the upper lower back and exiting the body at the side of the scapula. He managed to tear the vessel wall of the right common carotid artery, causing an arterial bleed, which led to a haemorrhagic shock and death.

Little is known about the clinical history of patients, committing suicide by a power drill. Byard was the first to thoroughly evaluate the clinical history of the patient that revealed a long-standing psychiatric disorder and a change in a pharmacotherapy few days before the suicide event [2]. In our case, it was possible to obtain a complete medical history which revealed the presence of alcohol dependency in combination with a personality disorder and epilepsy. It is well known that psychiatric disorders are an important risk factor for suicide and are described as an underlying condition in up to 90% of all suicides in several world regions [16]. People with epilepsy also have a higher rate of suicide ideation and suicide attempts than the general population, especially in cases of psychiatric comorbidity with a significantly higher incidence of suicide and suicide attempts associated with substance use [17]. Therefore, the deceased from our presented case was at a very high risk for suicidal behaviour. However, despite showing clear signs of self-harm, there is no record of him being asked about suicidal thoughts or intents after he did not continue with his yearly appointments with the psychiatrist, although he was often treated for other medical conditions. Cases like this should raise awareness of the importance of a holistic approach to patient treatment.

## Key points

1. Power drill injuries are extremely rare and when self-inflicted they are almost always connected to underlying psychiatric disorder(s).
2. Death following power drill injuries is often not instantaneous and sometimes requires multiple attempts to cause significant damage to internal organs.
3. Unplanned complex suicide can involve unusual methods with an intent to finish the suicidal act after previously used more conventional but unsuccessful methods.
4. In order to identify potential suicidal behaviour or intent, a more holistic approach to patient care is needed when patients exhibit clear signs of underlying psychiatric disorder(s).

## Data Availability

Not applicable.
